# Morphometric Analysis of Mandibular Ramus Indices for Sex Prediction Using Digital Orthopantomograms (OPGs) and the GNU Image Manipulation Program (GIMP) Software

**DOI:** 10.7759/cureus.93520

**Published:** 2025-09-29

**Authors:** Harshal R Thube, Vina Vaswani, Neelam Chandwani, Manish Shrigiriwar

**Affiliations:** 1 Forensic Medicine, All India Institute of Medical Sciences, Nagpur, IND; 2 Forensic Medicine, Yenepoya Medical College (Deemed to be University), Mangalore, IND; 3 Dentistry, All India Institute of Medical Sciences, Nagpur, IND

**Keywords:** forensic anthropology, gimp software, mandible, morphometry, orthopantomogram, sex determination

## Abstract

Background: Sex determination forms a crucial part of human identification in forensic medicine, anthropology, and clinical practice. The mandible is a durable and morphologically dimorphic bone, making it valuable for sex estimation. This is crucial in the forensic investigation. Advances in digital radiography and open-source software, such as the GNU Image Manipulation Program (GIMP), allow precise morphometric analysis, potentially enhancing the accuracy of forensic investigations.

Aim: The study aimed to predict sex by calculating mandibular morphometric indices from digital orthopantomograms (OPGs) using the GIMP software in a central Indian population.

Materials and methods: This cross-sectional study analyzed 500 archival OPGs (250 males and 250 females aged 20-40 years) obtained from the Department of Dentistry, AIIMS Nagpur. Four mandibular parameters, including condylar ramus height, projective ramus height, maximum ramus breadth, and minimum ramus breadth, were measured bilaterally using the GIMP software under double-blind conditions.

Results: All four mandibular parameters showed statistically significant sexual dimorphism (p < 0.001), with males displaying larger linear dimensions than females. All four parameters showed significant dimorphism, with condylar ramus height and maximum ramus breadth contributing most strongly to sex prediction. The mean condylar ramus height was 58.89 mm in males and 52.36 mm in females; projective ramus height was 53.73 mm vs. 47.94 mm; maximum ramus breadth was 31.80 mm vs. 27.42 mm; and minimum ramus breadth was 25.55 mm vs. 23.12 mm. Regression analysis confirmed significant differences after adjusting for age. Discriminant function analysis demonstrated that condylar ramus height and maximum ramus breadth contributed most strongly to the sex classification.

Conclusion: Mandibular morphometric indices obtained from OPGs using the GIMP software are reliable indicators of sex, with significant sexual dimorphism in the ramus dimensions. This cost-effective and reproducible method has the potential to enhance forensic identification protocols, especially where DNA or conventional methods are unavailable.

## Introduction

Any human identity connotes unique biological traits such as body structure, facial features, complexion, and genetic makeup, which distinguish each individual. Human identity is routinely established using traditional documentation or simple fingerprinting techniques. However, when questions arise about establishing identity in certain conditions, such as mass disasters, criminal offenses, legal disputes, and humanitarian forensics, scientific techniques are required apart from civil documents. The consideration of determining age, gender, race, and stature by scientific methods narrows down the individual’s identity. Among these, sex determination is essential in various fields, such as forensic medicine, anthropology, and clinical medicine. The skull, pelvic bone, and cranium are essential skeletal tools for identification [1]. In the case of skeletonized remains or decomposed human bodies, traditional methods of sex identification often involve visual and manual assessment of skeletal remains, which can be subjective and inconsistent. With advancements in digital radiography and image analysis, it is now possible to obtain precise measurements of any human bone morphometric index, potentially improving the accuracy and objectivity of sex prediction methods.

Various authors have attempted gender determination in a specific population from different skeleton parts, such as the femur [2,3], patella [4,5], mandible [6], occipital condyle [7], and sternum [8].

The challenge of applying findings from a particular group, particularly concerning skeletal elements such as the cranium and mandible, to other groups has heightened the focus on conducting population-specific research for sex determination using these elements [9]. The human mandible, or lower jawbone, is one of the most diversified bones in the skeleton and can be beneficial in determining the sex of an individual.

Morphometric analysis of different parameters of the jaw bone by observing the orthopantomogram (OPG) of an individual can be correctly performed using the freely available software GNU Image Manipulation Program (GIMP) 2.10.34 (revision 3) [10,11]. While handling mass disasters such as floods, earthquakes, tsunamis, building collapses, and fire incidents, or while performing an autopsy on an exhumed dead body, or in case of a decomposed body or a dismembered body, the facial features may be beyond identification. In such scenarios, a simple OPG radiograph can detect the sex of an individual with good accuracy.

Similarly, the freely available GIMP software can play an important role because of its precise prediction and ability to correct image errors. Digital radiography has advantages in decomposed facial features; without further dissection, the exact morphometric dimensions of facial structures can be obtained. The present study aimed to predict sex by calculating mandibular morphometric indices from digital radiographs using GIMP software.

## Materials and methods

This cross-sectional study was conducted at the Department of Forensic Medicine, AIIMS Nagpur. Samples were collected from the records of the Department of Dentistry at AIIMS, Nagpur. A total of 500 samples, comprising 250 males and 250 females aged 20-40 years, were randomly selected using standard digital OPGs. The study included cases where the sex was known and OPG radiographs were available for each sample, with distinct and clearly visible head alignment, high image quality and sharpness, full permanent dentition, and no radiographic signs of trauma. Radiographs with artifacts and pathological conditions of the bones, such as the mandible, were excluded from the study. Each patient was exposed to a panoramic X-ray using the Papaya Genoray system (Genoray Co., Ltd., Seongnam, South Korea) at 74 kV, 12 mA for 14.3 seconds. Subsequently, the OPG images were selected and uploaded to the GIMP software.

The parameters to be observed were measured using the GIMP software 2.10.34 (revision 3) [11]. The following parameters were measured bilaterally using this software. Three observers repeated each reading thrice to eliminate bias, and the average value was included in the analysis. The two observers were unaware of the sex of the individual OPG, maintaining a double-blind condition. Additionally, GIMP software offers the benefit of eliminating magnification bias and minimizing measurement bias.

A) Condylar ramus height: This refers to the vertical distance from the condyle to the most prominent point on the lower edge of the ramus. B) Projective ramus height: This is the vertical distance from the condyle to the bottom edge of the bone. C) Maximus ramus breadth: This is the horizontal span from the foremost to the rearmost point of the ramus. D) Minimum ramus breadth: This is the narrowest horizontal distance between the front and back points of the ramus.

**Figure 1:**
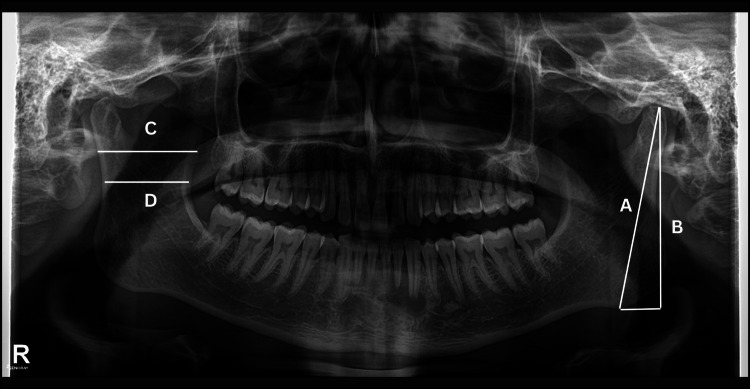
OPG demonstrating the linear measurements of mandibular parameters using GIMP GIMP, GNU Image Manipulation Program; OPG, Orthopantomogram

Figure 1 below shows the OPG, demonstrating the linear measurements of mandibular parameters mentioned with A, B, C, and D, using freely available GIMP software [11].

Measurement workflow in GIMP software

To maintain consistency, the following steps were performed for each OPG image: Step 1: After installing and opening the GIMP software, which is a freely available tool, we opened the OPG image from the folder. With minor adjustments, we adjusted the contrast and brightness. Care was taken to avoid distortion of the OPG image. The "Zoom" tool was then opened, and the desired side of the mandible was enlarged for precise tracing.

In the next step, the inbuilt scale marker on the OPG image was adjusted using the "Measure" tool. The tool converts the pixel measurements into millimeters by setting the scale under the image-scale-set image-scale prompt. Subsequently, the linear parameter was calculated using the "Measure" tool by placing the first cursor point on the most superior aspect and the second point at the most inferior prominent point on the mandible and recording the displayed distance. These measurements were entered directly into an Excel (Microsoft Corp., Redmond, WA) spreadsheet. These measurements were repeated to eliminate intra-observer bias. 

All tools used in this study, including measurement indices derived from OPG images (e.g., condylar ramus height, projective ramus height, and maximum and minimum ramus breadth), were calculated manually using the GIMP software [11]. No proprietary scoring systems or paid instruments were used. Data analysis was conducted using the jamovi (version 2.7.6, the jamovi project, Sydney, Australia), a freely available open-source statistical software package. An independent t-test was employed to compare the measurements between the groups, and discriminant function analysis was applied to the measurements.

## Results

The present study was conducted in the Department of Forensic Medicine and Toxicology. The archival data of OPG from the Department of Dentistry were carefully screened, and cases were selected after applying the above-mentioned inclusion criteria. These cases were then considered for the study. After taking measurements using the GIMP software, the data were entered in MS Excel format. Data analysis using jamovi yielded the following results, as shown in Table 1.

**Table 1:** Comparison of mandibular measurements between the two sexes SD, Standard deviation

S.N.	Parameter	Male (in mm)		Female (SD)		t-value	p-value
		Mean	SD	Mean	SD		
	Test used	Independent t-test					
1	Condylar ramus height	58.89	4.52	52.36	5.46	5.046	<0.001
2	Projective ramus height	53.73	5.13	47.94	5.36	4.274	<0.001
3	Maximum ramus breadth	31.80	2.51	27.42	2.55	6.705	<0.001
4	Minimum ramus breadth	25.55	2.74	23.12	2.76	3.422	<0.001

Table 1 presents various mandibular parameters measured on both the right and left sides for males and females, along with the average of both sides (R+L/2). The independent t-test is used for analysis. The statistical significance of the differences between males and females is indicated by the p-values, all of which are <0.001, suggesting highly significant sex-based differences for each parameter. Overall, this suggests that males tend to have larger linear measurements (e.g., ramus height and breadth), indicating a more robust mandibular morphology.

Table 2 summarizes the results of linear regression analysis of mandibular measurements with gender (female compared with male) as the predictor. Both the unadjusted regression coefficient and the adjusted regression coefficient are reported, along with their 95% confidence intervals. The results indicate highly significant sex differences in all variables, with the independent t-test used for comparisons.

**Table 2:** Linear regression analysis of outcomes with gender (female compared with male) for measurements of mandible *Age adjusted ARC, Adjusted regression coefficient; CI, Confidence interval; URC, Unadjusted regression coefficient

S.N.	Parameter	URC (95% CI)	p-value	t_Gender	ARC (95% CI)*	p-value	t_Age
		(R+L/2)			(R+L/2)		
	Test used	Independent t-test					
1	Condylar ramus height	-6.53 (-7.41, -5.65)	<0.001	-14.53	-6.53 (-7.41, -5.65)	<0.001	0.24
2	Projective ramus height	-5.78 (-6.7, -4.86)	<0.001	-12.29	-5.78 (-6.7, -4.86)	<0.001	0.27
3	Maximum ramus breadth	-4.38 (-4.82, -3.93)	<0.001	-19.34	-4.38 (-4.83, -3.94)	<0.001	-0.57
4	Minimum ramus breadth	-2.44 (-2.92, -1.95)	<0.001	-9.91	-2.44 (-2.93, -1.96)	<0.001	-0.79

Table 3 indicates the standardized coefficients of the six measurements. The most negative coefficients for maximum ramus breadth and the positive coefficients for minimum ramus breadth suggest their relative contribution to the discrimination between the male and female groups. From the above data, we obtained the following formula to separate the data according to sex: Sex- (-2.886*A2 + 0.936*B2 - 9.848*C2 + 7.178*D2 + 1.86*E2 + 3.762*F2).

**Table 3:** The standard canonical discriminant function coefficients

Function coefficient	Function	Z_perm
Test used: Discriminant function analysis with permutation testing		
Condylar ramus height	-2.886138208980608	-4.716203495149280
Projective ramus height	0.9360562395271091	-1.4513471589302700
Maximum ramus breadth	-9.847683841987752	-46.623319958194700
Minimum ramus breadth	7.1780240995108375	-36.28520115641930

Table 4 presents the standardized and unstandardized coefficients from the multiple linear regression analysis of mandibular parameters. The results demonstrate that maximum ramus breadth and condylar ramus height exert strong negative effects, while minimum ramus breadth makes a strong positive contribution to sex classification.

**Table 4:** The standardized and unstandardized coefficients

Parameter	Standardized coefficient	Unstandardized coefficient	t-value	p-value
Test used: Multiple linear regression analysis				
Condylar ramus height	-2.886	-1.23	-6.12	<0.001
Projective ramus height	0.936	0.41	2.89	0.004
Maximum ramus breadth	-9.848	-2.87	-8.77	<0.001
Minimum ramus breadth	7.178	2.12	7.31	<0.001

**Figure 2:**
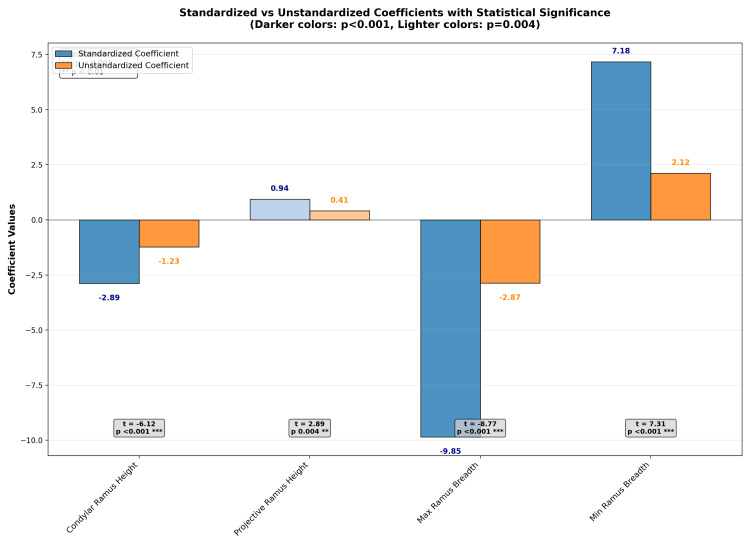
A visual comparison of the standardized and unstandardized coefficients, along with the t-values and p-values, annotated above each bar

Figure 2 shows a visual comparison of the standardized and unstandardized coefficients, along with the t-values and p-values, annotated above each bar.

## Discussion

This study aimed to predict sex using mandibular morphometric indices derived from digital OPGs and GIMP software. The findings revealed notable sexual dimorphism across all assessed mandibular parameters, with males typically exhibiting larger linear dimensions and females exhibiting greater angular measurements. These findings are largely consistent with those of previous studies on mandibular sexual dimorphism.

A) Condylar and projective ramus heights: The study found these to be significantly larger in males, aligning with the findings of More et al. and Arthanari et al [12,13]. This reinforces the reliability of the ramus height as an indicator of sex. B) Maximum and minimum ramus breadths: These were also larger in males, supporting earlier studies, such as Verma et al. [14], which highlighted the ramus breadth as a sexually dimorphic feature.

Gender-based differences

The data show that several craniofacial parameters, such as condylar ramus height, projective ramus height, maximum ramus breadth, and minimum ramus breadth, were significantly smaller in females than in males. All p-values in the table are less than 0.001, indicating that the differences observed for each craniofacial parameter between males and females were statistically significant. These findings align with earlier studies, such as those by Kambylafkas et al. and Gaur et al., which have established that mandibular ramus dimensions, particularly height and breadth, are reliable indicators of sexual dimorphism [15,16]. The condylar height emerged as the most dimorphic variable, corroborating the observations by Humphrey et al. and Vats et al., who noted that the vertical parameters of the ramus exhibit greater sexual dimorphism than the angular parameters [17,18]. This might be attributed to the longer duration of mandibular growth in males under the influence of testosterone, which leads to larger and more robust mandibles.

Clinically, these data can be used to understand the distinct differences in craniofacial structures between males and females, which might be relevant for understanding sexual dimorphism in craniofacial morphology.

Regression analysis consistently revealed significant differences between females and males across various craniofacial parameters, underscoring the importance of considering sex when evaluating craniofacial structures. Our method uniquely uses the GIMP software, a freely available image processing tool, for linear morphometric assessments on digital radiographs. This methodology not only democratizes access to forensic measurement tools but also offers consistent and reproducible results when measurements are calibrated properly. Similar approaches have been adopted in resource-limited settings by many authors, where open-source tools have demonstrated promising accuracy.

Comparison with standard textbooks such as Krogman’s “The Human Skeleton in Forensic Medicine” and Bass’s “Human Osteology” further validated our anatomical landmarks and choice of indices. Both texts emphasize the robustness and forensic reliability of the mandibular ramus, especially when the skull is fragmented or unavailable for analysis [19,20].

Discriminant function

The canonical discriminant function allows classification of individuals into groups based on their craniofacial measurements. The coefficients suggest which variables are more influential in distinguishing between the groups. A higher discriminant function score corresponds to a higher likelihood of belonging to a particular group (likely females, based on positive coefficients), and a lower discriminant function score corresponds to the other group (likely males) [21].

Application of canonical discriminant analysis

This analysis is useful in fields such as forensic anthropology, where distinguishing between male and female remains based on craniofacial measurements is important. The canonical discriminant function provides a mathematical model for separating individuals into sex categories based on their craniofacial features. These coefficients can guide clinicians and researchers in identifying patterns of craniofacial growth, sexual dimorphism, and variability among populations. They can also be used for personalized treatment in orthodontics and maxillofacial surgery, where such anatomical features may play a role in treatment planning.

The use of the GIMP software for measurements represents an advancement over previous methods. While earlier studies, such as those by More et al. and Ojha et al., used Kodak Master View and ImageJ, respectively, GIMP offers the advantage of being freely available and potentially reducing measurement bias [12,22]. Several dental image software options can be used for measurement and analysis in forensic odontology, including DentaScan, ImageJ, ModelMatch3D, and Kodak Master View. These tools aid in tasks such as comparing antemortem and postmortem images, odontometric analysis, and 3D model comparison. These are sophisticated, paid software with high efficiency in image analysis for diagnosis and treatment purposes. However, forensic odontology requires only base-level image analysis, which can be effectively performed using freely available software such as the GIMP. 

The overall accuracy of sex prediction in this study (not explicitly stated but implied to be high given the significant differences) appears comparable to or better than that of some previous studies. For instance, More et al. reported a 69% accuracy using mandibular ramus measurements [12].

The strengths of this study include its age range (20-40 years) and sample size (500), providing a robust dataset for analysis. This improves upon earlier studies with smaller sample sizes or wider age ranges, which could introduce age-related variations.

Linear regression analysis further validated sexual dimorphism, showing significant differences even after adjusting for age. This addresses a limitation of previous studies that did not consider age-related changes in mandibular morphology. However, this study had some limitations. It focused on a specific population, which may restrict the generalizability of the findings to other ethnic groups. Additionally, the use of two-dimensional radiographs, though practical and widely accessible, may not fully capture the three-dimensional complexity of mandibular morphology that CT scans can provide, as demonstrated by Suzuki et al. [23]. Furthermore, the predictive model was not tested on an independent validation set, which would have enhanced the robustness and reliability of the results.

This study contributes valuable data to the field of forensic anthropology and sex determination by leveraging digital technology to enhance traditional morphometric methods. These findings reinforce the sexual dimorphism of the mandible and demonstrate the potential of freely available software for forensic analysis.

## Conclusions

In conclusion, this study on predicting sex through the analysis of mandibular morphometric indices using digital radiographs and the GIMP software has significant implications for forensic anthropology and dental science. The analysis of measurements such as condylar and projective ramus height and maximum and minimum ramus breadth demonstrated a correlation between these specific mandibular dimensions and sexual dimorphism, suggesting that these morphometric indices can serve as reliable indicators of biological sex when other methods are unavailable or impractical. The use of digital radiography enhances the efficiency and accuracy of measurements, further supporting the utility of this approach in both clinical and legal contexts. Future investigations should aim to expand the sample size and incorporate diverse populations to validate these findings, thus enriching our understanding of mandibular variations and their potential applications in sex estimation.
